# Altered Cerebrospinal Fluid (CSF) in Children with Ataxia Telangiectasia

**DOI:** 10.1007/s12311-020-01175-x

**Published:** 2020-08-19

**Authors:** S. Woelke, R. Schrewe, H. Donath, M. Theis, M. Kieslich, R. Duecker, G. Auburger, R. Schubert, S. Zielen

**Affiliations:** 1 Department for Children and Adolescents, Division of Allergology, Pulmonology and Cystic Fibrosis, Goethe University, Theodor-Stern-Kai 7, 60590 Frankfurt/Main, Germany; 2 Department for Children and Adolescents, Division of Pediatric Neurology, Goethe University Hospital, Frankfurt, Germany; 3 Experimental Neurology Medical Faculty, Goethe University, Frankfurt, Germany

**Keywords:** Ataxia telangiectasia, Cerebrospinal fluid, Cytokines, Albumin ratio, Neurodegeneration, Ataxia score

## Abstract

Ataxia telangiectasia (A-T) is a devastating multi-system disorder characterized by progressive cerebellar ataxia and immunodeficiency. The neurological decline may be caused by multiple factors of which ongoing inflammation and oxidative stress may play a dominant role. The objective of the present investigation was to determine cerebrospinal fluid (CSF) proteins and possible low-grade inflammation and its relation to age and neurological deterioration. In the present study, we investigated 15 patients with A-T from 2 to 16 years. Our investigation included blood and CSF tests, clinical neurological examination, A-T score, and MRI findings. The albumin ratio (AR) was analyzed to determine the blood–brain-barrier function. In addition, inflammatory cytokines (IL-1α, IL-6, IL-8, IL-12 p40, IL-17A, IFN-γ, TNF-α) were measured by the multiplex cytometric bead array. We compared the results with those from an age-matched control group. Three of the A-T patients were analyzed separately (one after resection of a cerebral meningioma, one after radiation and chemotherapy due to leukemia, one after stem cell transplantation). Patient had significantly more moderate and severe side effects due to CSF puncture (vomiting, headache, need for anti-emetic drugs) compared with healthy controls. Total protein, albumin, and the AR increased with age indicating a disturbed blood barrier function in older children. There were no differences for cytokines in serum and CSF with the exception of IL-2, which was significantly higher in controls in serum. The AR is significantly altered in A-T patients, but low-grade inflammation is not detectable in serum and CSF.

## Introduction

Ataxia telangiectasia (A-T) is a devastating human autosomal recessive disorder with genetic instability. A-T is characterized by cerebellar degeneration and oculocutaneous telangiectasia, accompanied by immunodeficiency, recurrent respiratory infections, growth retardation, and cancer predisposition [1–4]. The patients have an expected lifespan of only 20–30 years, due to respiratory failure and cancer [5]. The prevalence of A-T is highly variable and estimated by various authors at approximately 1:40,000–1:400,000.

Disease progression of A-T is demonstrable at different organ levels of which neurological decline is the most prominent feature of disease [1, 6]. Most patients with classical A-T are forced into wheelchair around puberty. A-T represents a striking combination of tissue-specific degenerative processes and a complex clinical phenotype. Neurological decline may be caused by multiple factors of which inflammation and oxidative stress play a dominant role [7–9]. The underlying mechanisms of disease progression are based on lack of major ATM functions. It is well known that Ataxia-Telangiectasia-Mutated-(ATM)-negative cells (neuron, lung, and liver cells) are unable to counteract inflammation and oxidative stress. In this perspective, ATM acts as a sensor of oxidative stress. Indeed, elevated serum IL-6 and IL-8 levels in patients with A-T have been described and may contribute to the disease progression [10–15]. How inflammation and neurodegeneration interact is still a matter of ongoing debate.

The neuropathological changes underlying the cerebellar motor findings in A-T show a macroscopically visible cerebellar atrophy with a thinning of cerebellar hemispheric foliae and cerebellar vermis, which result from a reduction in Purkinje cells, granular cells and basket cells [6, 16, 17]. In addition, a recent study from our group showed that ApoB excess and reelin signaling deficits reflect the neurodegeneration in A-T in a sensitive and specific way [18]. Imaging studies repeatedly demonstrated cerebellar atrophy in all patients with classic A-T [19, 20]. Interestingly MRI-imaging studies in younger patients with A-T do not show any signs of cross abnormalities or signal alterations [own data unpublished]. Several rating scales for A-T in order to monitor the course and severity of cerebellar symptoms have been proposed [21, 22]. Unfortunately, the proposed rating scales for A-T are of limited value due to vague evaluation of test items in the first years of life [6, 21]. The neurological decline may be caused by multiple factors of which ongoing inflammation and oxidative stress may play a dominant role [7, 8, 10, 11]. Thus, there is an unmet need of new surrogate markers of neurodegeneration and disease progression. So far, only two studies (one from our group) used cerebrospinal fluid (CSF) to search for potential biomarker candidates of neural atrophy in A-T [18, 23]. Both studies, the one in adult and our recent study in children, applied a high-throughput LC/MS-based label-free protein quantification technology to characterize proteins in CSF samples and to identify differentially expressed proteins, which can serve as biomarker candidates. The objective of the present investigation was to determine CSF proteins and possible low-grade inflammation and its relation to age and neurological deterioration. Therefore, we analyzed clinical complaints after spinal tap, as well as blood and CSF for presence of inflammatory cytokines by the multiplex cytometric bead array (CBA) in A-T patients and healthy controls. In addition, results were compared with the clinical neurological examination, the A-T score and the magnet resonance imaging (MRI) findings.

## Materials and Methods

### Patients

In the period between July 2010 and March 2013, 15 patients with A-T were recruited for the study. All patients were clinically and/or genetically diagnosed with A-T according to recent WHO recommendations [24]. The study was approved by the responsible ethics committee at the Goethe University Frankfurt (application number 296/09). The study was conducted following the ethical principles of the Declaration of Helsinki, regulatory requirements, and the code of Good Clinical Practice, and informed consent was obtained from all parents and patients. The trial was registered at ClinicalTrials.gov (NCT02285348).

Inclusion criteria for A-T patients:

- Diagnosis of A-T (clinical or genetic)
- Age < 18 years
- Agreement to take the samples by blood sampling and lumbar puncture
- No clinical or laboratory evidence of severe infection with central nervous system (CNS) involvement
- Leukocytes < 12,000/μL
- CRP < 2 mg/ dL
- No recent CNS surgery
- No other CNS damage
- No CNS malignancy
- No stem cell transplantation

Three of the 15 patients with an A-T were excluded from the evaluation of the so uncomplicated A-T group due to the exclusion criteria mentioned earlier. In detail, there was one patient after resection of a cerebral meningioma, one suffered from seizures after accidental irradiation of the brain suffering from acute leukemia since the diagnosis of A-T was not established at that time, and one patient who was recently treated by stem cell transplantation. All three patients (A-T identification number 13–15) showed morphologic clear cerebral structural changes at the MRI, which would have falsified the measurements for the uncomplicated A-T group (Table 2). To analyze the influence of age, the healthy patients were divided into two clinical groups: six patients who were under 10 years old and six patients older than 10 years. In addition, there was a consecutive numbering with increasing age (identification number A-T-01–A-T-12).

The control group consisted of non-A-T patients receiving lumbar puncture due to clinical indication. The indications consisted of the exclusion of meningitis, therapeutic punctures in pseudotumor cerebri, diagnosis of facial nerve paralysis, suspected Lyme disease, headache and seizures. There were 12 patients; 8 were male and 4 female. The age distribution was an average of 9 years and 8 months. Again, there was a consecutive numbering with increasing age (identification number K-01–K-12). Table 1 shows the clinical characteristics of patients and the control group:

### Lumbar Punctures

The lumbar punctures were performed according to the present clinical standard and as far as possible with atraumatic puncture needles (SPROTTE® 21G needles). In the applied puncture technique, the muscular layer is first pierced above the spinal canal by means of an introducer, via which the a traumatic CSF needle is then inserted. This reduces the complication rate of CSF [25, 26].

In all cases, due to the existing psychomotor deficit and strong anxiety of the patients, the lumbar punctures were performed in deep conscious analgesic sedation using low doses of midazolam and Es-ketamine. The patients were monitored during the study by ECG, blood pressure measurement, and oxygen saturation. The procedure was performed in the presence of a pediatric intensive care physician. In eight cases, the lumbar puncture was performed following a routine cMRI in deep analgesic sedation. In these cases, analgesic sedation and circulatory monitoring was performed by the colleagues of the Anesthesiology Clinic of the Goethe University Frankfurt.

In general, 2–3 ml CSF transferred to three tubes was taken regardless of age or body weight. One part of the CSF was analyzed routinely for cellular content, albumin, protein, lactate, and glucose; two vials were stored and frozen at – 80 °C until final measurement.

All patients were bed-rested for 12–24 h after lumbar puncture and received crystalloid infusion solutions as well as pain and anti-emetic agents as needed. Possible side effects were recorded as follows:

- No side effects
- Mild headache, nausea, vomiting
- Moderate to severe headache requiring analgesia, nausea or vomiting with the need for anti-emesis treatment

### Blood and CSF Analysis

Alpha Feto-protein (AFP), C-reactive protein (CRP), total protein, albumin, albumin CSF/serum ratio (AR), lactate, and glucose were determined from the blood serum and CSF in patients and controls. Since albumin is produced only outside the brain, it is a good marker for other proteins that leak from the blood into the cerebrospinal fluid, and thus for blood–brain-barrier integrity. After measuring the albumin in serum and CSF, the AR was calculated, in which the influence of the individual protein concentration in the serum on the CSF concentration is mathematically compensated. Higher AR values indicate a disturbed blood–brain-barrier function.

The concentrations of inflammatory cytokines (IL-1α, IL-6, IL-8, IL-12 p40, IL-17A, IFN-γ, TNF-α) were analyzed via CBA (BD Bioscience-PharMingen, San Diego, CA, USA) using an enhanced sensitivity setting with detection of cytokines at femtogram levels, as described [26].

### Neurological Assessment

The A-T patients were also examined by a pediatric neurologist as recently described [6, 20]. Disease progression was classified according to KAS [27]. Gait ataxia, standing ataxia, ataxia of the upper and lower extremity, dysarthria, intention tremor, and dysdiadochokinesis are assessed. Impairment is evaluated in points from 0 (nonexistent) to 5 (maximum deficiency) so that the maximum score is 35 points.

### MR Imaging

Patients underwent standardized MRI analysis of the brain, including T1-, T2-, and fluid-attenuated inversion recovery (FLAIR) sequences. An evaluation of cerebellar atrophy, brain stem, and basal ganglia as well as cervical was done by an experienced pediatric neurologist. Atrophy was scored semi-quantitatively as non-discernible (−), obvious (+), moderate (++), and severe (+++) as described recently [20] (Table 2).

### MRI Index

Two standardized sections were determined in the transversal plane in the T2-weighted sequences in order to measure the cerebrum in the transverse diameter. For the MRI Index, a quotient (diameter of cerebellum (b)/diameter of cerebrum (c)) was formed to obtain a comparability of the size ratios.

### Statistical Analysis

For statistical analysis, GraphPad Prism 5.01 (GraphPad Software) and Microsoft Excel were used. Values are presented as arithmetic means with SDs. For comparisons between the two study groups, Student’s unpaired *t* test was applied. Correlations were analyzed by Spearman’s or Pearson’s correlation coefficient. *P* values ≤ 0.05 were considered significant.

## Results

As shown in Table 1, we enrolled 15 A-T patients, 9 were male and 6 were females, but only 12 were analyzed as “uncomplicated” A-T group according to the inclusion and exclusion criteria. The age distribution was median 10.8 range (2.6 to 16.2) years. We compared the patients’ data of the so-called 12 healthy A-T patients with those of 12 healthy control subjects (median age 7.1 years; range, 0.2–16.9; male/female ratio 8:4 among 12 individuals) (Table 1). The A-T scores were significantly correlated with the age of the patients and with disease duration (correlation coefficient of 0.85, *p* < 0.001); as expected, more older patients were bound to wheelchair.

**Table 1:** Patients’ characteristics

	**Controls** ***n*** **= 12**	**A-T** **< 10 years** ***n*** **= 6**	**A-T** **≥ 10 years** ***n*** **= 6**	**A-T**^**a**^ ***n*** **= 3**	***P*** **value**
Age (years)		**5.1** (2.6–10.0)	**14.15** (11.6–16.2)	**9** (4.8–16.6)	0.001
Sitting in a wheelchair (*n*)	NA	**None**	***n*** **= 5**	***n*** **= 2**	0.01
KAS (points)	NA	**14.5** (13–22)	**22.5** (22–25)	**17.0** (6–27)	< 0.001
AFP (ng/dL)	**< 10.0**	**185** (30.7–501)	**485** (175–639)	**261** (105–709)	0.01
CRP (mg/dL)	**0.32** (0.01–2)	**0.04** (0–0.7)	**0.07** (0–0.2)	**0.32** (0.15–0.6)	n.s.
Protein in liquor (mg/dL)	**209.5** (119–276)	**163.5** (146–254)	**417.5** (174–588)	**447** (161–707)	0.002
Albumin in liquor (mg/dL)	**121.5** (71.6–155)	**100.5** (84.3–199)	**312** (112–403)	**282** (101–467)	0.004
Albumin quotient	**3.2** (1.76–4.8)	**2.5** (1.96–3.26)	**7.1** (2.87–8.8)	**7.6** (2.5–10.4)	0.01
Glucose in liquor (mg/dL)	**62** (51.5–77.1)	**59.7** (55.8–66.2)	**68.4** (59.6–78.6)	**51.8** (51.6.70.9)	n.s.
Lactate in liquor (mg/dL)	**1.42** (1.15–1.72)	**1.46** (1.15–1.61)	**1.6** (1.31–2.15)	**1.3** (1.09–1.82)	n.s.

Median (bold) and range are shown

*KAS* Klockgether ataxia score, *AFP* Alpha Feto-protein, *CRP* C-reactive protein, *n.s.* not significant, *NA* not available

^a^3 patients with complications and significant findings at MRI (one had a cerebral meningioma, one suffered from seizures after accidental irradiation of the brain, and one was recently treated by stem cell transplantation

### Side Effects of Spinal Puncture

**Fig. 1:**
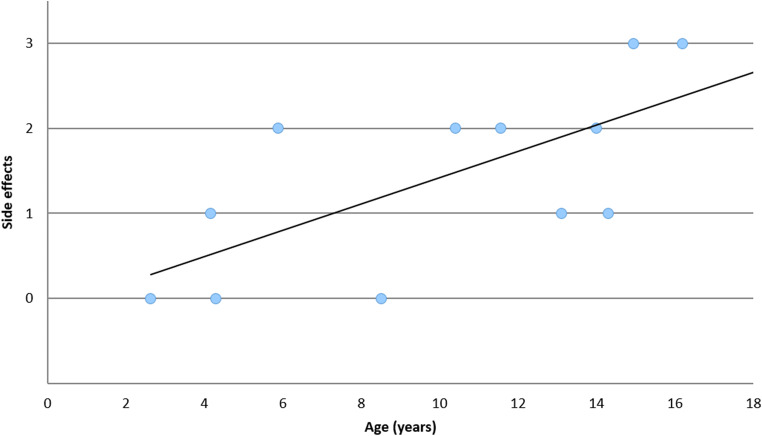
Side effects of CSF puncture in A-T patients in relation to age. 0 = none; 1 = mild; 2 = moderate; 3 = severe side effects

In the present study, side effects of CSF puncture occurred in 9 of 12 (75%) A-T patients in the sense of a post-puncture syndrome, and 6 (50%) of them even moderate to severe side effects. In the control group, there were only three (25%) cases of mild side effects in terms of headache and nausea. Three among the 12 patients showed no symptoms due to the lumbar puncture (Fig. 1). Thus, the A-T patients had significantly more adverse events compared with the control group (*p* = 0.001). In addition, older patients more often showed moderate to severe side effects of lumbar puncture (Spearman correlation coefficient 0.68, *p* = 0.014). The high rate of side effects was the reason that the study was terminated earlier than planned and no adult patients were included.

### Blood and CSF Findings

Serum levels of CRP, AFP, lactate, and glucose are shown in Table 2. There were no significant differences in cell count, lactate, and glucose, whereas total protein and albumin was significantly (*p* = 0.002) elevated in CSF compared with healthy controls as shown in Table 1. Interestingly, total protein and albumin were significantly correlated to age as recently described [18].

**Table 2:** MRI findings and A-T score

Patient number	Age (years)	Sex M/F	Ataxia Score (points)	Cerebellar atrophy vermis	Cerebellar atrophy hemispheres	Pontine atrophy	Spinal atrophy
1	2.6	M	13	−	−	−	−
2	4.2	F	14	−	−	−	−
3	4.3	M	16	++	+	−	−
4	5.9	F	14	−	−	−	−
5	8.5	M	15	++	++	−	−
6	10.0	F	22	n.d.	n.d.	n.d.	n.d.
7	11.6	M	25	++	++	+	+
8	13.1	M	22	−	−	−	−
9	14.0	F	23	+	+	−	−
10	14.3	M	22	++	++	+	−
11	14.9	M	25	−	−	−	−
12	16.2	F	20	++	++	+	−
13	4.8	M	6	+	−	−	−
14	9.0	M	17	++	++	−	−
15	16.6	F	27	++	++	+	+

Patients 1–6 were < 10 years; patients 7–12 > 10 years, and patients with complications, 13–15. Extent of neurodegeneration in MRI measured as described (20)

*n.d.* not determined

The AR of the A-T patients was significantly elevated (median 4.69 ± 2.66) compared with controls (median 3.07 ± 0.91). The AR increased with age, which correlated well with the protein in the CSF (correlation coefficient 0.85, *p* = 0.004).

In the control group, two CSF samples were found, with cell counts slightly above the limit of 10 cells/μL, but in each case, meningitis was ruled out. The standard value for pediatric patients is no more than 6–10 cells/μL in CSF (28). However, there was a trend for higher CRP levels of controls compared with A-T patients (*p* = 0.06). In addition, CRP levels of controls and patients showed a significant correlation to IL-6 levels in CSF (Spearman *r* = 0.4614; *p* = 0.02).

### Cytokines

**Fig. 2:**
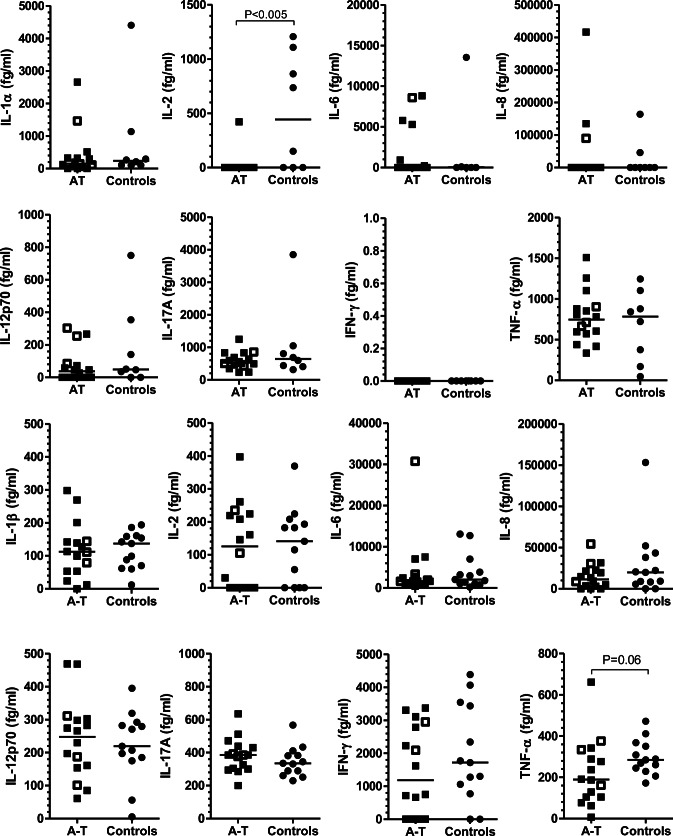
**a** Inflammatory cytokines and chemokines in serum. There were no differences between patients and controls for cytokines (IL-1α, IL-6, IL-8, IL-12 p40, IL-17A, IFN-γ, TNF-α) except for IL-2, which was significantly lower in A-T patients. Three A-T patients with complications and significant findings at MRI are displayed as open squares. **b** Inflammatory cytokines and chemokines in CSF. There were no differences between patients and controls for cytokines (IL-1α, IL-2 IL-6, IL-8, IL-12 p40, IL-17A, IFN-γ, TNF-α). A-T patients had lower levels of TNF-α. Three patients with complications and significant findings at MRI are displayed as open squares

We investigated the CSF versus serum in 15 children with A-T for inflammation. Overall, we found no differences in cytokine concentrations between patients and controls in serum and CSF with the exception of lower serum IL-2 in A-T patients versus controls with nominal significance, and a trend toward lower TNF-alpha levels in CSF (Fig. 2a, b).

### MRI Findings and A-T Score

**Fig. 3:**
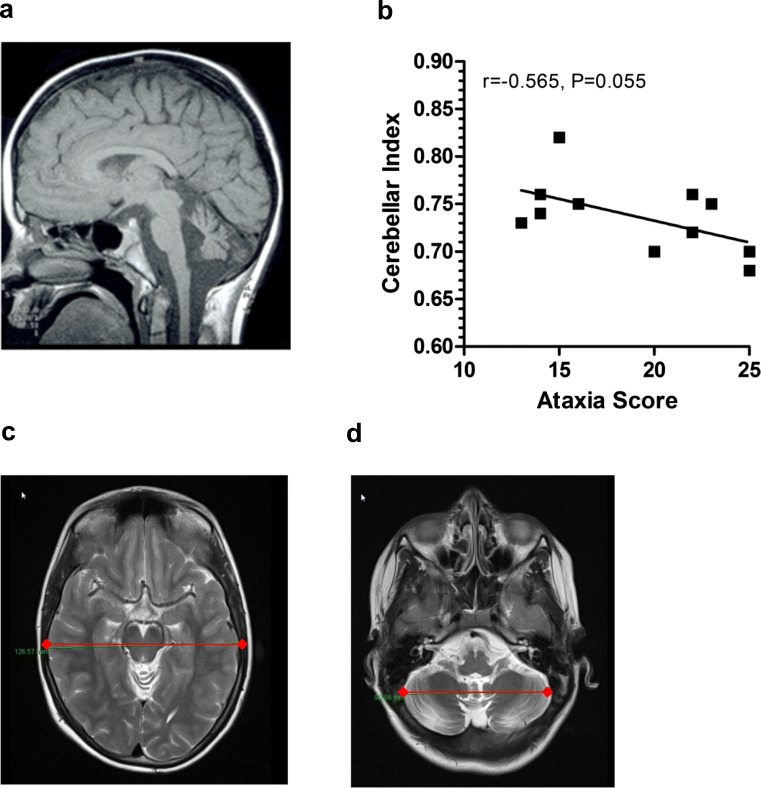
Imaging of the neurodegeneration in A-T patients. Cerebellar atrophy (**a**). Two standardized sections were determined in the transversal plane in the T2-weighted sequences in order to measure the cerebrum in the transverse diameter. For the MRI Index, a quotient ((diameter of cerebellum) (**b**)/(diameter of cerebrum) (**c**)) was formed to obtain a comparability of the size ratios. MRI Index was correlated to ataxia score (**d**)

In order to image the neurodegeneration pattern, MRIs of the patients were examined. Differences between patients below versus above 10 years of age are shown in Table 2. Two standardized sections were determined in the transversal plane in the T2-weighted sequences in order to measure the cerebellum/cerebrum in the transverse diameter. For the MRI Index, a quotient (diameter of cerebellum/diameter of cerebrum) was formed to obtain a comparability of the size ratios. Quotients from a minimum of 0.68 to a maximum of 0.82 were observed, with a mean value of 0.74. Comparison of the A-T score with the MRI Index almost reached significance *p* < 0.055, giving an idea of the shrinking cerebellum in A-T patients below the age of 18 years, with no adult patients being included (Fig. 3). However, there was no correlation between MRI Index and CSF findings (protein, albumin, and albumin ratio), most likely since the numbers (*n* = 11) were too small.

## Discussion

A-T is a devastating systemic disease characterized by neurodegeneration, increased risk for cancer, immunodeficiency, and failure to thrive. Disease progression and neurological decline are related to dysfunctional control of ROS and ongoing low-grade inflammation observed in the absence of the ATM disease protein [8–10, 12]. Our investigation is the first investigation into the tolerability of CSF puncture and into inflammatory proteins during childhood with A-T. The following important findings have to be discussed:

1. CSF puncture was not well tolerated in A-T patients.
2. Total protein, albumin, and AR were elevated in CSF, indicating a disturbed blood–brain barrier and/or ongoing cell degeneration, as recently described by our group [18].
3. There were no differences for cytokines in serum and CSF between A-T and controls of sufficient significance to withstand a statistical correction for multiple testing, such as Bonferroni.

The lumbar punctures were performed according to the present clinical standard with atraumatic puncture needles to reduce the rate of unwanted side effects. However, it should be noted that the overall complication rate in pediatric patients is higher than in adults [25, 26, 28, 29]. A common explanation is that the amount of CSF and the production rate are significantly lower, especially in smaller children, so that the loss of CSF from the total quantity of CSF is disproportionate. This leads to increased symptoms like headache, nausea, and vomiting of the post-puncture syndrome.

Although intravenous rehydration treatment was used in all patients after lumbar puncture to reduce these symptoms, there was a high rate of mild to moderate side effects in patients with A-T (A-T 75% vs. controls 25%). It was of great concern that 50% of the older patients had severe and long-lasting symptoms despite intravenous rehydration and anti-emetic treatment. This prompted us to terminate the study earlier than planned, although serious complications such as meningitis, nerve injuries, or seizures did not occur in any of the patients studied.

The very high rate of side effects was unexpected and is difficult to explain. One contributing factor may be the marked reduced nutritional state present in elderly patients with A-T, which is caused by the progressive neurological manifestations of dysarthria, muscular hypotonia, and swallowing difficulties [3, 4].

Another relevant point may lie in the fact that volume loss followed by rehydrating infusions would trigger a reduction of colloidal osmotic pressure within the brain ventricles. It is already known that hypo-osmotic as well as hyper-osmotic cell stress activates the ATM protein [30–32], thus signaling at the cell and organism level to correct the salt and protein concentrations that are crucial for osmotic homeostasis and for proper neuronal function. In A-T patients, the rapid recovery of normal osmotic pressure would be impaired by the pathogenic mutations in the ATM protein. Similarly, the pressure in blood vessels is regulated in an ATM-dependent manner [33], a mechanism that may contribute to the age-progressive dilatation of capillaries as a characteristic sign of this disorder. Young and lean people in general even without A-T suffer from an increased rate of side effects after lumbar puncture, as already described in the literature [25, 29]. To what extent neurodegeneration and shrinking of the cerebellum are related to these side effects cannot be further clarified. The observed significant correlation between age and severity of the patients with the severity of the side effects provides evidence for a significant influence of these anatomical changes on CSF function.

The increase of CSF total protein, albumin, and albumin ratio would normally be interpreted as evidence for blood–brain-barrier impairment, as often observed during inflammatory processes or meningeal carcinomatosis, given that albumin is not synthesized in brain but imported after synthesis in peripheral organs such as liver [34]. However, it is important to remember that the organism of A-T patients induces highly elevated levels of alpha-fetoprotein (AFP) as a diagnostic feature, and albumin synthesis might be selectively induced by a parallel mechanism. AFP and albumin are members of the same protein family [35]. Both serve to maintain colloidal pressure and vascular tone, AFP during fetal life, and albumin during adult life [36]; they are encoded by neighbor genes whose proximity is conserved on human chromosome 4/mouse chromosome 5/rat chromosome 14 [37], and both genes share a partially joint promoter/enhancer regulation of their expression [37, 38]. Thus, both factors are downregulated by elevated colloidal osmotic pressure [36], and the parallel expression regulation of both factors in response to epidermal growth factor, phorbol ester, or retinoic acid stimulation and butyrolactone-I treatment was demonstrated, with AFP responding much more sensitively [36, 39, 40]. After birth, the expression of AFP is downregulated and substituted by albumin expression with a more complex regulation during adult life [38, 41], an alternate system that is reflected by reports that butyrate and thyroid hormone show converse expression effects on the two transcripts [40, 42]. Thus, in several monogenic ataxia disorders it was observed that AFP is abnormally high while albumin is too low [43, 44], but the levels of total CSF protein and of CSF albumin increase until old age [45, 46]. Thus, the increase albumin levels might either be due to abnormal ATM-dependent regulations of osmotic pressure, or reflect premature aging, rather than being evidence for impaired blood–brain-barrier function and a possible neuroinflammatory process. Furthermore, albumin as the most abundant protein has a crucial role as main extracellular antioxidant and nutrient transporter [47]. Its antioxidant capacity mainly relies on its Cys34 residue that can be transformed into more oxidized forms, preventing the oxidation of other entities [48]. CSF albumin comes mainly from plasma, where it is synthesized by the liver in its native form, although it may also be directly originated in the microglia [49, 50]. After secretion and under an oxidative environment, albumin can be transformed into more oxidized forms, having an important impact on albumin distribution and metabolism [50]. Thus, the increased albumin CSF levels might reflect a role to compensate the oxidative stress that was observed in the cerebellum of A-T patients.

*Atm*-deficient mice and individuals with A-T show elevated ROS and ongoing low-grade inflammation as a key component of A-T pathology [10–15]. Furthermore, it was shown that ibuprofen had anti-inflammatory and anti-oxidative effects in ATM-deficient conditions [51]. In addition, the use of dexamethasone was able to ameliorate the neurological symptoms in A-T patients [51, 52].

However, our data in serum and CSF cannot confirm recent reports of ongoing mild inflammation in patients with A-T [11–13, 15]. This is difficult to explain. Maybe a more pronounced inflammation is demonstrable only in older patients. In support of this idea, we could recently show that C-reactive protein (CRP) was significantly correlated with age in a larger cohort of patients [4]. Alternatively, the innate immune system in A-T appears to be hypersensitive to exogenous challenges [11, 13, 53], and we may have missed such an inflammatory signal in our “uncomplicated A-T patients” during investigation. Maybe, if we would follow our patients for a longer time, elevation of IL-6 and IL-8 would have been demonstrable since a study in mice found that brain IL-6 expression was greatest in the cerebellum of mice that received peripheral endotoxin [54]. This prompted us to correlate CRP and IL-6 levels in both controls and A-T patients, and interestingly a significant correlation was established. This is in line with the reports of parents that neurological symptoms of their children get worse during an infection episode. The low levels of IL-2 in serum are explained by the well-known T-cell deficiency of A-T patients [55, 56].

The present study has some limitations. The number of patients who underwent CSF punction was very small since the study was earlier terminated due to severe side effects. In addition, no adults were included, thus more advanced disease stages were not investigated.

It can be criticized that our controls suffered from suspected meningitis and therefore the cytokine levels were measured not in healthy children. This view is in accordance with slightly higher TNF values in CSF and CRP levels in controls than A-T patients. However, it was ethically not possible to sample CSF from healthy children. Consequently, it is extremely difficult to establish normal values of CSF. Therefore, the question has to be addressed how to define a normal control CSF. In order to achieve an almost “normal” CSF, we used CSF for further analyses only of those controls who had no significant elevation of leukocytes and CRP.

## Conclusion

This is the first study which showed that A-T patients had significantly more moderate and severe side effects due to CSF puncture compared with healthy controls. In CSF total protein, albumin and the albumin ratio (AR) significantly increased with age. There were no differences for cytokines in serum and CSF, most likely why our patient group was small and only few older patients were included.

## Abbreviations

AR: Albumin ratio
A-T: Ataxia telangiectasia
AFP: Alpha Feto-protein
*ATM*: Ataxia telangiectasia mutated
CBA: Cytometric bead assay
CRP: C-reactive protein
CSF: Cerebrospinal fluid
ECG: Electrocardiogram
IFN-γ: Interferon-gamma
IL-1α: Interleukin-1 alpha
IL-6: Interleukin-6
IL-8: Interleukin-8
IL-12 p40: Interleukin-12 p40
IL-17A: Interleukin-17A
KAS: Klockgether ataxia score
WHO: World Health Organization
ROS: Reactive oxygen species
ATM: Ataxia telangiectasia mutated
TNF-α: Tumor necrosis factor alpha
